# Quantitative and qualitative analysis on the legislative production relating to healthcare in passage in the National Congress in the years 2007 and 2008

**DOI:** 10.1590/1516-3180.2013.1316576

**Published:** 2013-12-01

**Authors:** Viviane Cristina dos Santos, Marcos Bosi Ferraz

**Affiliations:** I MD. Former Researcher at São Paulo Center for Health Economics and Researcher in the Health Economics Research Group, Universidade Federal de Minas Gerais (UFMG), Minas Gerais, Brazil; II Professor and Director, São Paulo Center for Health Economics and Adjunct Professor, Department of Medicine, Escola Paulista de Medicina - Universidade Federal de São Paulo (EPM-Unifesp), São Paulo, Brazil

**Keywords:** Health, Policy making, Health policy, Legislation as topic, Decision making, Saúde, Formulação de políticas, Política de saúde, Legislação como assunto, Tomada de decisões

## Abstract

**CONTEXT AND OBJECTIVES:**

The Federal Constitution of 1988 allowed the National Congress to contribute towards formulation of new public policies. The objective of this study was to analyze the legislative production that dealt with health issues that was in passage in the National Congress between January 2007 and December 2008.

**DESIGN AND SETTING:**

Descriptive-exploratory cross-sectional study with quantitative and qualitative approaches, conducted in a federal university.

**METHODS:**

The analysis material for the study comprised 144 draft bills that were classified and subsequently randomly evaluated by 155 professionals working within the healthcare system.

**RESULTS:**

The analysis showed that the Workers' Party (PT) and Brazilian Democratic Movement Party (PMDB) were the parties that presented the largest proportions of the draft bills (12.5% and 11.1%); 25.4% of the draft bills were presented by congress members with academic qualifications within healthcare and only 1.4% of the draft bills became transformed into legal regulations. In questionnaire responses, 51.5% of the evaluators did not consider the draft bills to be viable, 40.6% did not consider them to be relevant and 52.5% said that if the draft bills were not approved it would not be harmful to Brazilian society.

**CONCLUSION:**

In analyzing the data from this study, it was noted that the legislative production relating to healthcare was low and the transformation rate from draft bill to legal regulation was negligible. The results from the evaluation showed that the quality of legislative production was impaired.

## Abstract (pt)

**CONTEXTO E OBJETIVOS:**

A Constituição Federal de 1988 possibilitou ao Congresso Nacional contribuir na formulação de novas políticas públicas. O objetivo do estudo foi analisar a produção legislativa que trata de questões de saúde, em tramitação no Congresso Nacional, entre janeiro de 2007 a dezembro de 2008.

**TIPO DE ESTUDO E LOCAL:**

Estudo descritivo-exploratório com corte transversal e abordagem quantitativa e qualitativa, conduzido em uma universidade federal.

**MÉTODOS:**

O material de análise do estudo foi constituído por 144 projetos de lei (PLs), classificados e posteriormente avaliados, aleatoriamente, por 155 profissionais do sistema de saúde.

**RESULTADOS:**

A análise mostrou que o Partido dos Trabalhadores (PT) e o Partido do Movimento Democrático Brasileiro (PMDB) foram os partidos que mais apresentaram PLs (12,5% e 11,1%), 25,4% dos PLs foram apresentados por parlamentares com formação na área da saúde e apenas 1,4% dos PLs foram transformados em norma jurídica. Em relação aos questionários, 51,5% dos avaliadores não consideraram os PLs viáveis, 40,6% não consideraram os PLs relevantes e 52,5% disseram que a eventual não aprovação do projeto de lei não seria prejudicial para a sociedade brasileira.

**CONCLUSÃO:**

Ao analisar os dados deste estudo, percebeu-se que a produção legislativa no âmbito da saúde foi baixa, a transformação dos projetos de lei em norma jurídica foi mínima, e os resultados da avaliação mostraram que a qualidade da produção legislativa está comprometida.

## INTRODUCTION

In order to understand how the legislature has been working to contribute towards a more democratic political process relating to healthcare, it is necessary to study the legislative process and its results in the light of the needs of the healthcare sector. Thus, it is necessary to understand how the legislature is organized.

The 1988 Brazilian Constitution, in title IV,^1^ chapter I, provides that legislation should be enacted by the National Congress, which is composed of the Chamber of Deputies and the Federal Senate. The Chamber of Deputies is composed of representatives of the people and the Federal Senate is composed of representatives of the states and the Federal District. The legislative function exercised by the National Congress that will be examined in this study is the drafting of laws. The initiative for introducing laws can be taken by the Chamber of Deputies, the Federal Senate, the President of the Republic, the Federal Supreme Court, the Superior Courts, the Attorney General of the Republic or citizens, in the manner set forth in the 1988 Constitution.

The procedure for assessing legislative matters that are in progress is bicameral (i.e. it takes place in both legislative chambers). Thus, one chamber starts the process and the other one reviews it; except in cases of private matters of each chamber. Upon approval, the bill is forwarded to the President to sanction or veto it. This procedure is followed for all complementary and ordinary bills.

The Chamber of Deputies and the Senate have standing and temporary committees covering different fields and areas of activity. The functions of these standing committees include discussion of and voting on proposals that are subject to decisions made in plenary sessions of the chambers, and discussion of and voting on such decisions for which, according to the regulations of the chamber, the jurisdiction of the full chamber is waived. In the Senate, the standing committee responsible for social security is known as the Social Affairs Committee,^2^ while in the Chamber of Deputies such issues are addressed by the Social Security and Family Committee.^3^

Since this study will discuss the Brazilian legislation that addresses healthcare, it is necessary to clarify what the 1988 Constitution, in Articles 196 and 197, stipulates in this regard.

Article 196: Healthcare is everyone's right and a duty of the State, and it shall be ensured through social and economic policies that aim to reduce the risk of illness and other health hazards and provide universal and equal access to actions and services for promotion, protection and recovery of health.

Article 197: Healthcare actions and services are of public importance, and it is incumbent upon the Government to provide, in accordance with the law, for regulation, supervision and control; these actions shall be carried out directly or by third parties and also by individuals or private legal entities.^1^

Thus, it can be seen that the Federal Constitution of 1988 not only gave a new shape to healthcare in Brazil, but also provided Congress with the opportunity to participate in and contribute towards formulation of new public policies. According to Figueiredo and Limongi,^4^ the Federal Constitution of 1988 restored the legislative powers that had been curtailed through successive constitutional reforms imposed by military governments, and also, in several respects, increased its powers in relation to the Constitution of 1946.

## OBJECTIVE

To quantitatively and qualitatively describe and analyze the legislative production addressing health issues that was under ongoing discussion in congressional committees (Social Security and Family in the Chamber of Deputies; and Social Affairs in the Senate) in the years 2007 and 2008.

## METHODS

This was an exploratory and descriptive cross-sectional study with quantitative and qualitative approaches. The study consisted of two distinct phases: in the first phase, we conducted a quantitative survey by means of stratification, classification and analysis of bills of law. In the second phase, a qualitative approach was taken, using a questionnaire containing closed questions that was applied to a group of professionals working within the healthcare system, in order to tabulate and evaluate the relevance, feasibility, strategic alignment and possible impacts of the proposed bills.

The analysis material for the study consisted of bills relating to healthcare that were in passage in the Senate and in the Chamber of Deputies, which were filed in the respective legislative chambers between January 2007 and December 2008. Because of the large number of legislative matters that were in Congress, we chose to assess the propositions that were within the standing committees relating to healthcare. Thus, to identify bills, a survey was conducted on the Chamber of Deputies' website, in the Social Security and Family Committee^5^ and on the Senate's website, in the Social Affairs Committee.^6^

This survey to determine the material for analysis was conducted in January 2009. It found 509 proposals in the Chamber of Deputies and 169 in the Senate, which were in passage with filing dates in the years 2007 and 2008.

The following criteria were used to select the material found:

a. Inclusion criteria: As a general criterion, we selected only the bills in passage that related healthcare actions and services. As specific criteria, we selected the bills in passage within the jurisdiction of the Brazilian National Health System (Sistema Único de Saúde, SUS) or the Ministry of Health, which related to healthcare professionals, health insurance and actions that would have an impact on the healthcare financial system.

b. Exclusion criteria: Other propositions were excluded from the research (i.e. draft legislative decrees, petitions and proposals for regulation, control, reporting, messages, representation, complaints and official letters), along with matters relating to social security.

In accordance with these selection criteria, out of the 509 proposals found to be in passage in Congress, 440 were bills and 69 were other propositions (requirements and proposals for regulation, control, reporting, representation, complaints and official letters). Among the 440 bills, 112 bills in the Chamber of Deputies were identified as material for analysis in the present study. In the Senate, out of the 169 bills found, 32 bills (26 bills that originated in the Senate and six that originated in the Chamber of Deputies) were identified as material for analysis in this study.

After obtaining approval for the research project that resulted in the present study, from the Research Ethics Committee of Universidade Federal de São Paulo (Unifesp), we conducted qualitative and quantitative analysis on the material gathered. The quantitative analysis was done by means of classification that followed the criteria: procedural rules; origin of the presentation of the bills, stratification of the bills by year; region of the country and political party of the congressman who filed the bill, healthcare classification of the bill, disease and specialty group, focus on private or public healthcare, status of bill (filed, approved or in passage), and budgetary nature.

The qualitative analysis was conducted through evaluation of the bills using a questionnaire containing closed and open questions that was answered by a group of professionals working within the healthcare system. The aim was to tabulate and evaluate the relevance of the bill (importance of the proposals for the healthcare system), its viability (capacity for implementation in relation to infrastructure and costs), its strategic alignment (synchronization of the proposals with government policies and guidelines) and its impact (assessment of risks and consequences resulting from failure of the bill).

The questionnaire was prepared and divided into three parts: Part I (questions about the participants' demographic and professional characteristics); Part II (questions about the professionals' opinions in relation to the Brazilian healthcare system and legislature); and Part III (questions evaluating the bill under analysis). Thus, each professional answered Parts I and II of the questionnaire only once. Part III of the questionnaire was answered as many times as the number of bills assessed, i.e. one questionnaire for each bill.

The group of evaluators was heterogeneous: professionals from diverse academic backgrounds, different areas of professional practice and different regions of the country who were familiar with the healthcare system and/or healthcare policies. The analysis was blind, i.e. the bills were not identified with any number or with the author's name, in order to avoid any possible influence on the evaluation process.

Methodologically, it was defined that each of these professionals would evaluate three bills and give responses to the questionnaire. Likewise, each bill would be rated by three people. Thus, at least 144 evaluators would be needed. We chose to identify 188 people (30% more), to allow for any need for replacements. The evaluator selection process was defined according to sectors of representation, in order to comply with the methodology defined, i.e. diversified academic background and distinct professional areas. Thus, nine groups of evaluators were chosen, with approximately 21 people in each of the following entities: 1. pharmaceutical industry, 2. private healthcare services, 3. public healthcare services, 4. regulatory agencies, 5. healthcare operators, 6. Ministry of Health and departments, 7. diagnostic analysis laboratories, 8. non-governmental organizations/civil society entities/patient associations, and 9. trade associations/unions.

In order to identify 188 professionals to participate in the study and answer the questionnaire, we chose to look for representatives of the nine groups set up above, so as to get a list with names of possible participants.

A "Letter of Invitation" providing a link to access the research project on the internet and an individual password was sent out by email. By accessing the link, the evaluator came to a homepage containing a text giving information about the study, explanations about the questionnaire and about the bills available for analysis, and a free and informed consent statement. After the evaluator had answered the questionnaire that was available on the website, the results were entered into the research database.

The original intention was that each bill would be rated by three people, but among the 177 people who responded to the questionnaire, 22 people responded only to Parts I and II, i.e. they did not respond to Part III of the questionnaire relating to the evaluation of the bills. We chose not to take these data into consideration. Thus, 155 people evaluated the bills. Among the 144 bills, one bill (0.7%) was evaluated by only one person, 22 bills (15.3%) were evaluated by two people, 96 bills (66.7%) were evaluated by three people, 19 Bills (13.2%) were evaluated by four people and six bills (4.1%) were evaluated by five people. Descriptive statistical analysis was used to characterize the parameters studied.

## RESULTS

Tables 1, 2 and 3 present the characterization and description of the bills. Table 1 shows the characteristics that identified the bills.^7^ The criterion of "nature of procedure" was not used for the 26 bills that originated in the Senate, since this legislative chamber did not provide this information on its website. Thus, regarding this variable, 118 bills were evaluated (112 bills that were in the Chamber of Deputies and 6 bills that were in the Senate but originated in the Chamber of Deputies). Among these, 81.4% (96) followed an ordinary procedure, 17.8% (21) a priority procedure and 0.8% (1) an emergency procedure.

**Table 1:** Characteristics identifying the bills^7^

		n	%
Type of procedure in the chamber*	Ordinary	96	81.4%
	Priority	21	17.8%
	Urgent	01	0.8%
	**Total**	**118**	**100.0%**
Origin	Congressman	115	79.9%
	Senator	28	19.4%
	CPL	01	0.7%
	**Total**	**144**	**100.0%**
Submission date	Year 2007	83	57.6%
	Year 2008	61	42.4%
	**Total**	**144**	**100.0%**
Region	Southeast	51	35.7%
	South	29	20,2%
	Northeast	27	18.9%
	North	19	13.3%
	Center-west	17	11.9%
	**Total†**	**143**	**100.0%**
Political Party	PT	18	12.5%
	PMDB	16	11.1%
	PSDB	15	10.4%
	PSB	12	8.3%
	PP	11	7.6%
	PSC	09	6.2%
	PTB, PR, PPS, PDT, DEM‡	40	28.0%
	PRB, PHS, PCdoB‡	15	10.5%
	Others	08	5.4%
	Total	144	100.0%
Academic qualification of the congressmen who submitted the bill	Health field	37	25.7%
	Other fields	107	74.3%
	**Total**	**144**	**100.0%**
Situation in December 2009	In passage	135	93.7%
	Shelved	07	4.9%
	Converted into legal rules	02	1.4%
	**Total**	**144**	**100.0%**

* No information relation to type of procedure was found for the 32 bills presented in the Senate

† One bill was submitted by the Committee on Participatory Legislation (CPL), and therefore this bill does not have a corresponding region

‡ The political parties that presented the same numbers of bills have been grouped

PT = Workers' Party

PMDB = Brazilian Democratic Movement Party

PSDB = Brazilian Social Democracy Party

PSB = Brazilian Socialist Party

PP = Progressive Party

PSC = Social Christian Party

PTB = Brazilian Labour Party

PR = Party of the Republic

PPS = Popular Socialist Party

PDT = Democratic Labour Party

DEM = Democrats

PRB = Brazilian Republican Party

PHS = Humanist Party of Solidarity

PCdoB = Communist Party of Brazil.

**Table 2:** Topics of the bills^7^

		n	%
Health*	Health promotion	10	5.4%
	Disease prevention	45	24.3%
	Diagnosis	18	9.7%
	Treatment	67	36.2%
	Rehabilitation	10	5.4%
	This criterion does not apply	35	19.0%
	Total*	**185**	**100.0%**
Disease	Acute	24	16.7%
	Chronic	11	7.6%
	Both	66	45.8%
	This criterion does not apply	43	29.9%
	**Total**	**144**	**144.0%**
Medical specialty*	Medical clinic	75	50.7%
	Obstetrics	14	9.5%
	Cardiology	03	2.0%
	Hematology	04	2.7%
	Oncology	03	2.0%
	Neurology	04	2.7%
	Others	22	14.9%
	This criterion does not apply	23	15.5%
	Total*	**148**	**100.0%**
Approach*	Public healthcare system	101	57.7%
	Private hospitals/clinics	29	16.6%
	Healthcare plan operators	14	8.0%
	Healthcare professionals	13	7.4%
	Others	18	10.3%
	Total*	**175**	**100.0%**
Budgetary nature	Yes	06	4.2%
	No	138	95.8%
	**Total**	**144**	**100.0%**

* The topics used allowed more than one answer.

**Table 3:** Numbers of bills presented in the National Congress, numbers of senators and federal congressmen and numbers of bills presented by senators and federal congressmen per year and per region of the country^7^

Region of the country	Chamber of Deputies			Federal Senate		
	No. of bills (two years)*	No. of members†	Bills/member/year	No. of bills (two years)	No. of senators	Bills/senator/year
	n (%)	n (%)	n	n (%)	n (%)	n
Southeast	43 (37.4%)	179 (34.9%)	0.12	8 (28.6%)	12 (14.8%)	0.33
South	22 (19.1%)	77 (15.0%)	0.14	7 (25.0%)	9 (11.1%)	0.38
Northeast	20 (17.4%)	151 (29.4%)	0.06	7 (25.0%)	27 (33.3%)	0.13
North	17 (14.8%)	65 (12.7%)	0.13	2 (7.1%)	21 (26.0%)	0.05
Center-west	13 (11.3%)	41 (8.0%)	0.16	4 (14.3%)	12 (14.8)	0.16
**Total**	**115 (100%)**	**513 (100%)**	**0.11**	**28 (100%)**	**81 (100%)**	**0.17**

* One bill was presented by the Committee on Participatory Legislation (CPL)

† Using the list of congressmen who took office in 2007 (as stated on the website of the Chamber of Deputies).

Regarding the origin, 79.9% (115) of the bills were presented by Congressmen, 19.4% (28) by Senators and 0.7% (1) by the Committee on Participatory Legislation (CPL) (a forum through which organized civil society can intervene directly in the production system for rules and laws, through making suggestions for improvements to existing legislation or drafting of new rules).

Regarding the date of submission of the proposals, 57.6% of the bills selected were presented in 2007 and 42.4% (61) in 2008. The southeastern region presented the greatest number of projects (35.7%), and the northern (13.3%) and central-western regions presented the lowest numbers of projects (11%).

PT (12.5%) was the political party that presented most bills followed by PMDB (11.1%) and PSDB (Brazilian Social Democracy Party) (10.4%). Only 25.7% of the parliamentarians that presented bills relating to healthcare had academic qualifications in this field. Most of them (74.3%) had academic qualifications in other fields.

In a new survey conducted in December 2009, i.e. one year after gathering the analysis material for the study, it was found that most of the bills (93.7%) were still in passage, (4.9%) had been shelved and only (1.4%) had been converted into legal rules.

Table 2 presents the topics of the bills, i.e. the main health-related topics addressed in the bills selected. In a group named Health, it could be seen that bills addressing the topic of treatment of diseases accounted for 36.2%, followed by prevention (24.3%). Among the 144 bills selected, this characterization did not apply to 19.0% because these bills addressed issues targeting healthcare professionals or the pharmaceutical industry, or were of budgetary nature, among other topics covered. Most of the bills (70.1%) could be categorized in a group named Acute/Chronic Disease, but this criterion did not apply to the other 29.9%. The latter included bills relating to pregnancy (prevention and diagnosis).

In characterizing the bills according to medical specialty, half of the bills (50.7%) related to clinic medicine, because the focus was on the health-disease process, without any approach within a specific specialty. On the other hand, obstetrics was the specialty that appeared most often in the matters discussed in the bills.

Through a thematic group named Approach, it was sought to ascertain what the focus of the health-related bills was. Thus, more than half of the bills (57.7%) were directed towards the public healthcare system, 16.6% towards private hospitals and clinics and 8% towards health plan operators. In addition, 7.4% of the bills targeted healthcare professionals, through addressing issues relating to training, professional practice and working hours, and 10.3% focused on other areas such as pharmaceuticals, medical supplies and food companies. In relation to bills of budgetary nature, only 4.2% of the bills targeted healthcare financial mechanisms.

According to the survey data, the region that presented the largest number of bills was the southeast, followed by the south and the northeast. Table 3 shows, by region of the country, the number of bills presented in each legislative chamber and the number of congressmen for each region of the country.

From analysis on the health-related legislative production of the Chamber of Deputies, it could be seen that the southeastern region presented the largest number of bills. However, by correlating the regional representation of the members in the Chamber of Deputies and the number of bills presented by region, it can be seen that the region with the highest production of health-related bills was the central-western region, with 0.16 bills/member/year, followed by the southern region with 0.14 bills/member/year and the northern region with 0.13 bills/member/year.

In the Senate, the region with the highest production of health related bills was the southern region, with 0.38 bills/senator/year, followed by the southeastern region, with 0.33 bills/senator/year, and the lowest rate was found in the northern region, with 0.04 bills/senator/year.

Tables 4 and 5 relate to the qualitative analysis on legislative production. In this evaluation on the 144 bills, 155 people participated in the survey and answered the questionnaire. Among the participants in this evaluation, more than half (59.4%) were male, but there was a significant participation of females (40.6%), and the predominant age group was from 31 to 60 years old (89.6%). Just over one third of the participants had a medical degree, but more than half of them were not practicing medicine as their main professional activity. Professionals from different academic fields also participated: 80.6% had a lato sensu postgraduate degree and 25.8% had a stricto sensu postgraduate degree. The main function and professional activity practiced by the respondents (i.e. the evaluators of the bills) was in administration or management. There was participation from the public, private and third (voluntary) sectors. Regarding the representativeness of the respondents according to region of the country, there was participation from all regions of the country, but predominantly from the southeast (61.9%).

**Table 4:** Health-related bills evaluated by up to five respondents with regard to the characteristics of viability, relevance, strategic alignment, impact and results from voting on each bill presented^7^

		n	%
Viability			
Is the bill well defined and described, such that its implementation and execution is possible?	No	238	54.1%
	Yes	202	45.9%
	Total*	**440**	**100.0%**
Relevance			
Considering the actual needs and priorities of the country, in terms of healthcare, is the proposed bill relevant?	No	189	43.0%
	Yes	251	57.0%
	Total*	**440**	**100.0%**
Strategic alignment			
Is there any alignment between the bill and the country’s. Healthcare priorities and policies?	No	245	55.7%
	Yes	195	44.3%
	Total*	**440**	**100.0%**
Impact			
Would failure to pass this bill have any negative impact on and/or do any harm to Brazilian society?	No	244	55.5%
	Yes	196	44.5%
	Total*	**440**	**100.0%**
Voting on bill			
If there was a public consultation to decide whether this bill should become a law, what would your vote be?	Approval	253	57.5%
	Disapproval	140	31.8%
	Abstention	47	10.7%
	Total*	**440**	**100.0%**

* Each respondent evaluated up to three bills (155 respondents).

**Table 5:** Overall assessment of the proposals presented

Average rating given			Voting results regarding approval and non-approval		
Rate*	n	%	Voting†	n	%
Very poor (score 0 to 2.0)	13	9.0%	100% non-approval	19	13.2%
Poor (score 2.1 to 4.0)	27	18.8%	Up to 1/3 approval (1 to 33%)	29	20.1%
Fair (score 4.1 to 6.0)	59	41.0%	Up to 2/3 approval (34 to 66%)	53	36.8%
Good (score 6.1 to 8.0)	34	23.6%	Over 2/3 approval (67 to 99%)	09	6.3%
Excellent (score 8.1 to 10)	11	7.6%	100% approval	34	23.6%
**Total**	**144**	**100%**	**Total**	**144**	**100%**

* These data relate to the average scores for each of the 144 bills evaluated by up to five respondents, who gave scores between 0 (worst score possible) and 10 (best score possible)

† These data relate to the votes on 144 bills; first we measured the bills that got 100% approval votes and 100% non-approval votes (abstention and disapproval) and then we measured the bills that got up to 33%, 66% or 99% approval votes.

Initially, the respondents were asked for their personal opinions about the Brazilian healthcare system and the legislature. Regarding their level of satisfaction with the legislature, it was seen that more than half (54.2%) were dissatisfied, 23.9% were very dissatisfied, 19.3% were neither satisfied nor dissatisfied and only 2.5% were satisfied. In assessing the public healthcare system assessment, 41.8% of the respondents rated it as poor/very poor, 38.7% rated it as fair and 19.3% rated it as good. None of the respondents selected the excellent category. On the other hand, regarding the private healthcare system, only 9.6% considered the system to be poor/very poor, while 43.8% rated it as fair and 46.4% as good/excellent.

Table 4 shows the results from the evaluation questionnaire for each bill. The same bill was assessed by up to five people, and thus, cumulatively, there were 440 evaluations on 144 bills.

The first evaluation criterion used was "Viability". The participants were asked whether they believed, from reading the bill, that its manner of implementation and execution were clearly stated. More than half of the respondents (54.1%) responded that the ways of enabling the propositions were not well defined and described in the bill, while 45.9% answered that these were clear.

The second evaluation criterion was "Relevance." Regarding this category, the participants were asked whether the matter proposed in the bill was relevant for the Brazilian population, considering their real needs and priorities in terms of healthcare. More than half of the respondents (57%) answered yes and 43% said no.

In relation to "Strategic Alignment," we asked whether the bill was aligned with the country's priorities and healthcare policies: 55.7% answered no and 44.3% answered yes. Through the criterion "Impact," we tried to evaluate whether rejection of the bill would have a negative impact and/or would be detrimental to Brazilian society: 55.5% answered no and 44.5% answered yes. Another criterion used involved simulation of a public consultation, by asking the respondent to vote for or against the bill that he or she was analyzing. More than half (57.5%) would approve (vote for) the bill that they analyzed, 31.8% would dis-approve (vote against) and 10.7% would not make a choice (i.e. they would abstain).

Table 5 relates to the criterion "Overall rating of the bill." The respondents were guided to assign a grade to assess the bill under analysis, taking into consideration the other characteristics assessed: from 0 (worst score possible) to 10 (best score possible). Given that each bill was evaluated by up to five people, it was necessary to average the grades received for each bill evaluated. Thus, 31.2% of the bills were rated as good/excellent, 41.0% as fair and 27.8% as poor/very poor.

Table 5 also shows the public consultation, i.e. the personal opinion of each respondent through voting on the bill (for, against or abstention) that they analyzed. The criterion used to prepare this table was measurement of the bills that achieved 100% approval rates and 100% non-approval rates (abstention and votes against) among the respondents, i.e. only the bills for which all the respondents had the same opinion, bearing in mind that each bill was evaluated by up to five people. Out of the 144 bills, 36.4% received a unanimous vote, among which 34 bills (23.6%) received 100% approval and 19 bills (13.2%) received 100% non-approval.

## DISCUSSION

In analyzing the situation of the selected proposals, one year after data-gathering, it was seen that only two of the bills (1.4%) had been converted into legal rules. Both of these originated in the Chamber of Deputies: one under an emergency procedure and the other under an ordinary procedure. In a study presented to the Chamber of Deputies, Rodrigues^8^ argued that Brazilian legal production was much lower than the statistics seemed to indicate, since out of a total of 16,217 bills initiated by the legislature and first presented between 1989 and 1998, only 262 (1.62%) were converted into law. However, these data are insufficient to evaluate the performance of Congress.

Oliveira9 drew attention to "legislative inertia", noting that the average time taken to approve legislative proposals relating to education was 33 months. In relation to the situation of the proposals one year after the two-year period studied, it was noted that 93.7% of the bills selected were still in passage in one of the chambers and that 4.9% had been shelved. One limiting factor that impedes any review of this passage time is the possible obstacles to progress in the legislature, which this study did not evaluate but have already been discussed by other authors: Figueiredo and Limongi^4^ reported that the Executive power established the content of legal production and set the legislative agenda, thus leaving Congress unable to follow its own agenda. Rodrigues and Zauli^10^ showed that one obstacle that hindered the performance of Congress was indistinct use of editing and re-editing of provisional measures by the President. Santos^11^ argued that in the post-Constituent Assembly period, the Executive power influenced the legislative process of Congress, and that the larger the interests involved and the strategic importance of the players in action were, the smaller the chances of cooperation between the two powers would be. In a recent article, Baptista^12^ examined the legislative process relating to health issues and stressed that materials written by the legislature, without the support of the Executive, followed the slower procedures. Oliveira^13^ showed that there was a difference in procedural processes for converting propositions relating to school curricula into legal rules between those submitted by congressmen and those that proceeded in conjunction with the Executive's proposals. The first of these followed the ordinary procedure (average duration of four years) and the latter followed an emergency procedure (only taking five months).

Other important factors that should be highlighted are the low production of congressmen with an academic background in healthcare (25.7%) and the low performance of the Brazilian legislature regarding the healthcare sector (0.11 bills/congressman/year in the Chamber of Deputies and 0.17 bills/congressman/year in the Senate). On the other hand, the largest production of health-related bills came from congressman from the central-western region (0.16 bills/congressman/year) and from senators from the southern region (0.38 bills/senator/year).

The analysis on the results relating to approval or disapproval of the bills showed that only 23.6% of them were 100% approved by the evaluators. Moreover, regarding the general evaluation score given for each bill, only 31.2% had scores greater than 6.1. These results lead us to question whether there was any concern for quality in regulatory processes, or whether the concern was only for production of laws, because the results show that there were no criteria or planning in most of the bills proposed.

The analysis on the legislature showed that more than two thirds of the respondents (78.1%) were dissatisfied or very dissatisfied with the legislature. Aguiar and Valentin^14^ showed that the negative view of the legislature has worsened considerably over recent years, and that it has become an unpopular institution. The low legislative production and lack of institutional commitment have led to distrust of the legislative chambers and aversion to their members. This also revealed that, because of the paralysis of the legislative chambers, the judiciary had been asked to regulate matters that should be subject to legal texts.

Regarding the qualitative analysis on the material, some recent studies have discussed quality evaluation models for legislative production. According to Castro,^15^ concern about the quality of legislative production has become a matter of priority for many governments over recent decades, especially in Europe, because of the nefarious effects of legislation produced without planning and not in a careful manner. Proliferation of legislation with excessive quantities of rules has become an obstacle to its effectiveness over the years.

From a retrospective evaluation of legislation, Cristas^16^ proposed that analysis should be conducted on the three "E's": effectiveness or validity (to check the actual effects of the legislation in relation to compliance and implementation); efficiency (to assess the level of achievement of the objectives); and efficiency (to analyze the cost-benefit balance involved). Thus, a retrospective analysis can become prospective when it establishes improvements that can be made in the existing legislation. Soares^17^ highlighted that, as shown in European studies, the low quality of legislation in Brazil has an impact on gross domestic product (GDP) and other equally serious consequences, such as distrust of the effectiveness of laws, intense judicial activism and lack of credibility of institutions.

A program called "Better Regulation" was created in the European Union and priorities were set for the member states, such as: more systematic evaluation of the economic, social and environmental impacts of legislative initiatives; greater transparency in the legislative process; development of legislative simplification programs; and improvement of European legislation enforcement.^18^ In 2006, the European Commission established an expert group to evaluate member states' efforts relating to impact assessment and simplification of legislation. According to Soares,^17^ in Italy, the Chamber of Deputies created a standing committee in order to advise on the quality of legislative texts, in terms of uniformity, simplicity, clarity and propriety.

In analyzing the data from this study, and bearing in mind that the results represent a sample of only two years, it was noted that from a qualitative point of view, the legislative production relating to health issues was low. Congressmen with an academic background in healthcare (one fourth of the sample) produced little; and the rate of approval of legislative matters was negligible, since only 1.4% of the bills selected for this study were converted into legal rules. Regarding the quality of the bills, the results from the evaluation showed that the quality of the legislative production was compromised.

In this light, there is a need to create a culture in Brazil that places value on drafting and evaluating potential legislation, as exists in other countries. Good-quality legislative policies are necessary in the Brazilian regulatory system, so as to prevent uncontrolled reproduction of ineffective and unenforceable rules.

It is essential to conduct further prospective studies in order to study and evaluate the main causes that might explain the low quality of legislative production of Congress. Good-quality legislation (in terms of not only legislation but also regulation and legalization) is necessary to ensure efficiency and equity in Brazil, where the healthcare system has many needs and demands, but its resources are finite and scarce.

## CONCLUSION

Bearing in mind that the results represent a sample of only two years, it was noted that from a qualitative point of view, the legislative production relating to health issues was low. Congressmen with an academic background in healthcare (one fourth of the sample) produced little; and the rate of approval of legislative matters was negligible, since only 1.4% of the bills selected for this study were converted into legal rules. The quality of the legislative production was compromised.

## References

[1] Brasil. Senado Federal. Secretaria Especial de Editoração e Publicações. Subsecretaria de Ediçoes Técnicas. Constituição da República Federativa do Brasil (2010). Texto promulgado em 05 de outubro de 1988.

[2] Brasil. Congresso. Senado Federal (2007). Regimento Interno: Resolução no 93, de 1970. Texto editado em conformidade com a Resolução no 18, de 1989, consolidado com as alterações decorrentes de emendas à Constituição, leis e resoluções posteriores, até 2006.

[3] Brasil. Câmara dos Deputados (2000). Regimento Interno da Câmara dos Deputados.

[4] Figueiredo A, Limongi F (1995). Mudança constitucional, desempenho do legislativo e consolidação institucional. Revista Brasileira de Ciências Sociais.

[5] Brasil. Câmara dos Deputados. Comissão de Seguridade Social e Família http://www2.camara.leg.br/atividade-legislativa/comissoes-permanentes/cssf.

[6] Brasil. Senado Federal Comissão de Assuntos Sociais.

[7] Santos VC (2011). Análise qualitativa e quantitativa da produção legislativa relacionada à saúde em tramitação no Congresso Nacional nos anos de 2007 e 2008 [Qualitative and quantitative analysis of the legislative production related to health ongoing at the National Congress in 2007 and 2008] [dissertation].

[8] Rodrigues RJP (2000). Estudo comparativo sobre produção legislativa e remuneração parlamentar em países selecionados da Europa, América do Norte e América Latina.

[9] Oliveira RF (2005). O papel do Poder Legislativo na formulação das políticas educacionais [thesis].

[10] Rodrigues MMA, Zauli EM (2002). Presidentes e Congresso Nacional no processo decisório da política de saúde no Brasil democrático (1985-1998) [The presidents and the National Congress in the decision-making process of health policies in democratic Brazil (1985-1998). Dados.

[11] Santos MHC (1997). Governabilidade, governança e democracia: criação de capacidade governativa e relações executivo-legislativo no Brasil pós-constituinte. Dados.

[12] Baptista TW (2010). Análise da produção legislativa em saúde no Congresso Nacional brasileiro (1990-2006) [Analysis of health legislation in the Brazilian National Congress (1990-2006)]. Cad Saude Publica.

[13] Oliveira RF (2009). A agenda do Legislativo Federal para as políticas curriculares no Brasil (1995-2007) [The agenda of the Federal Legislative for curriculum policies in Brazil (1995-2007)]. Educ Pesq.

[14] Aguiar EA, Valentim MAA (2005). O poder legislativo, sua atuação e qualidade institucional [monograph].

[15] Castro AVJ (2007). Legística e modelos de avaliação legislativa: uma proposta para o aprimoramento da produção normativa municipal de Belo Horizonte.

[16] Cristas A (2006). Legística ou a arte de bem fazer leis. CEJ.

[17] Soares FM (2007). Legística e desenvolvimento: a qualidade da lei no quadro da otimização de uma melhor legislação. Revista da Faculdade de Direito da UFMG.

[18] Fraga A, Vargas A (2000). Da qualidade da legislação ou de como pode o legislador ser um fora-da-lei. Cadernos de Ciência de Legislação.

